# Cross-sectional study of health impairment related to post COVID-19 condition among participants of a large population-based cohort in Germany

**DOI:** 10.1038/s41598-025-07894-7

**Published:** 2025-07-16

**Authors:** Sophie Diexer, Jonas Frost, Peter Ahnert, Till W. Baernighausen, Hermann Brenner, Julia Fricke, Sabine Gabrysch, Karin Halina Greiser, Volker Harth, Jana-Kristin Heise, Rudolf Kaaks, André Karch, Thomas Keil, Bianca Klee, Carolina J. Klett-Tammen, Lilian Krist, Benedikt M. J. Lampl, Michael F. Leitzmann, Wolfgang Lieb, Claudia Meinke-Franze, Karin B. Michels, Ilais Moreno Velásquez, Nadia Obi, Annette Peters, Laura R. Pfrommer, Tobias Pischon, Oliver Purschke, Nicole Rübsamen, Tamara Schikowski, Börge Schmidt, Sigrid Thierry, Henry Völzke, Marvin N Wright, Hajo Zeeb, Rafael Mikolajczyk

**Affiliations:** 1 Institute for Medical Epidemiology, Biometrics and Informatics, Interdisciplinary Centre for Health Sciences, Medical Faculty of the Martin Luther University Halle-Wittenberg, Magdeburger Str. 8, 06112 Halle (Saale), Germany; 2 Institute for Medical Informatics, Statistics and Epidemiology, Universität Leipzig, Leipzig, Germany; 3 Heidelberg Institute of Global Health (HIGH), Medical Faculty and University Hospital, Heidelberg University, Heidelberg, Germany; 4 Department of Global Health and Population, Harvard School of Public Health, Harvard University, Cambridge, USA; 5 Africa Center for Health and Population Studies, Mtubatuba, South Africa; 6 Division of Clinical Epidemiology and Aging Research, German Cancer Research Centre (DKFZ), Heidelberg, Germany; 7 Institute of Social Medicine, Epidemiology and Health Economics, Charité – Universitätsmedizin Berlin, Berlin, Germany; 8 Municipal Health Strategies Unit, Public Health Department Breisgau-Hochschwarzwald/Freiburg, Freiburg im Breisgau, Germany; 9 Institute of Public Health, Charité – Universitätsmedizin Berlin, Berlin, Germany; 10 Research Department 2, Potsdam Institute for Climate Impact Research (PIK), Member of the Leibniz Association, Potsdam, Germany; 11 Division of Cancer Epidemiology, DKFZ Heidelberg, Heidelberg, Germany; 12 Institute for Occupational and Maritime Medicine Hamburg (ZfAM), University Medical Centre Hamburg-Eppendorf (UKE), Hamburg, Germany; 13 Department for Epidemiology, Helmholtz Centre for Infection Research, Brunswick, Germany; 14 Institute of Epidemiology and Social Medicine, University of Münster, Münster, Germany; 15 Institute of Clinical Epidemiology and Biometry, University of Würzburg, Würzburg, Germany; 16 State Institute of Health I, Bavarian Health and Food Safety Authority, Erlangen, Germany; 17 Regensburg Department of Public Health, Regensburg, Germany; 18 Institute of Epidemiology and Preventive Medicine, University of Regensburg, Regensburg, Germany; 19 Institute of Epidemiology, Kiel University, Kiel, Germany; 20 Institute for Community Medicine, University Medicine Greifswald, Greifswald, Germany; 21 Institute for Prevention and Cancer Epidemiology, Faculty of Medicine, Albert- Ludwigs-University Freiburg, Freiburg, Germany; 22 Molecular Epidemiology Research Group, Max-Delbrueck-Center for Molecular Medicine in the Helmholtz Association (MDC), Berlin, Germany; 23 Institute of Epidemiology, Helmholtz Zentrum München - German Research Centre for Environmental Health (GmbH), Neuherberg, Germany; 24 Chair of Epidemiology, Institute for Medical Information Processing, Biometry and Epidemiology, Medical Faculty, Ludwig-Maximilians-Universität München, Munich, Germany; 25 German Center for Diabetes Research (DZD e.V.), Neuherberg, Germany; 26 DZHK (German Centre for Cardiovascular Research), partner site Munich Heart Alliance, Munich, Germany; 27 German Center for Mental Health (DZPG), partner site Munich, Munich, Germany; 28 IUF-Leibniz Research Institute for Environmental Medicine, Düsseldorf, Germany; 29 Institute for Medical Informatics, Biometry and Epidemiology, University of Duisburg-Essen, Essen, Germany; 30 Universitätsklinikum Augsburg, Universität Augsburg, Augsburg, Germany; 31 Leibniz Institute for Prevention Research and Epidemiology - BIPS, Bremen, Germany; 32 Faculty of Mathematics and Computer Science, University of Bremen, Bremen, Germany; 33 Department of Public Health, University of Copenhagen, Copenhagen, Denmark; 34 Health Sciences Bremen, University of Bremen, Bremen, Germany

**Keywords:** Epidemiology, Epidemiology

## Abstract

Post COVID-19 condition (PCC) is a substantial burden for patients, society, and the healthcare system. Participants of the German National Cohort (NAKO) were asked in an online survey about their self-perceived health, symptoms related to PCC, and infection status. PCC was defined as reporting symptoms for the time window 4–12 months after infection. Of 110,375 respondents (73% response), 86,833 were included in this analysis. Of these, 44,451 (51%) did not report a SARS-CoV-2 infection (no infection), 26,726 (31%) reported an infection but no symptoms 4–12 months after infection (infection/no PCC), and 15,656 (18%) reported an infection and symptoms (PCC). The median number of current symptoms at the time of the survey was two for the "no infection" and the "infection/no PCC" group, and five for the "PCC" group. Participants with PCC had a substantially higher probability of having worse self-perceived health (OR 1.84, 95% CI [1.75; 1.93] compared to the "no infection" group, adjusting for sex, age, education and chronic diseases with elevated risk for developing PCC. After adjusting for the number of current symptoms related to PCC, this difference disappeared, suggesting that the symptoms collected explain the impairment of self-perceived health in the PCC group.

**Supplementary Information:**

The online version contains supplementary material available at 10.1038/s41598-025-07894-7.

## Introduction

Post COVID-19 condition (PCC) is a burden to the patients, the society and on the healthcare system^1^. PCC, as defined by the World Health Organization (WHO), describes new or persistent symptoms that are present at least 12 weeks after a SARS-CoV-2 infection and that cannot be explained by other causes^2^. Around 10–20% of SARS-CoV-2 infected individuals were estimated to be affected during the pandemic phase^3–5^. A number of symptoms are associated with PCC, and these symptoms can substantially impact individuals’ everyday functioning^6^.

Symptoms of individuals with PCC include, among others, fatigue, cognitive impairment, respiratory issues, and cardiovascular problems, which are often subjective and rely on self report^7,8^. However, many of these symptoms are not exclusive to PCC and also commonly observed in the general population, irrespective of a SARS-CoV-2 infection. For instance, fatigue was reported by almost 60% of a general Norwegian adult population in a survey^9^. A study in Denmark found that 50% of participants experience this symptom while it was 36% in a study in New Zealand^10,11^. Similarly, other symptoms attributed to PCC, such as cognitive difficulties or respiratory issues, are also widely reported among individuals without a history of COVID-19^10,11^. This overlap complicates the differentiation of PCC from general health impairment unrelated to SARS-CoV-2 infection.

In contrast, conditions like myalgic encephalomyelitis/chronic fatigue syndrome (ME/CFS), which share symptom similarities with PCC, are less prevalent. A study in the Netherlands indicated that 1% of the adult population experiences ME/CFS^12^.

Given this large spectrum between common conditions and severe impairment, our aim was to assess and compare the self-perceived health and symptom burden of individuals with and without PCC. Specifically, we analyzed three groups: individuals with no reported SARS-CoV-2 infection, individuals with a reported SARS-CoV-2 infection but not reporting symptoms afterwards, and individuals with a reported SARS-CoV-2 infection who subsequently fulfilled the definition of PCC. Additionally, we wanted to assess whether PCC also has a direct association with perceived health besides the measured symptoms. In other words, whether the difference in self-perceived health between those classified as having PCC or not can be explained by the investigated symptoms.

## Methods

### Study population

The present study used data from the German National Cohort (NAKO Gesundheitsstudie; NAKO). The design of the NAKO is extensively described in a series of publications^13–15^. In brief, NAKO is a population-based, prospective cohort study with 205,415 participants 19–74 years old at baseline. The participants were randomly selected from the compulsory city registries based on age and sex-stratified samples. Recruitment took place between 2014 and 2019 in 18 study centers across 16 regions in Germany. The participants were invited to comprehensive baseline on-site examinations at their local study center, were examined for the second time between 2019 and 2024, and are currently undergoing their third examination. In addition to these on-site examinations, an online survey with a focus on SARS-CoV-2 infections was sent to all NAKO participants who had provided an email address in autumn 2022. The questionnaire covered topics such as general health, SARS-CoV-2 infections and symptoms potentially linked to PCC. The online survey was described in detail elsewhere^16^.

The NAKO was approved by the ethical review committees of all 18 participating NAKO study centers using their own reference numbers. The study was conducted following the Helsinki Declaration and written informed consent was obtained from all participants.

### Measures

#### Sociodemographic variables, comorbidities, self-perceived health, and symptoms

In the NAKO baseline questionnaire, which was applied during on-site visits between 2014 and 2019, information on education was collected and recoded into the three categories based on the international Standard Classification of Education (ISCED-97)^17^. We also used data on chronic diseases from the NAKO baseline, focusing on diseases linked to an increased risk for developing PCC: anxiety and/or depression, asthma, chronic kidney disease, chronic obstructive pulmonary disease, diabetes, immunosuppression, and ischemic heart disease^18^. Participants had been asked, whether they ever had the specific disease.

In the 2022 online survey, participants provided information about their current health status using part of the 36-item Short-Form Health Survey (SF-36)^19,20^. In this study, we only used the question on self-perceived health in which participants are asked to rate their current health on a 5-point Likert scale from "Excellent" to "Poor". We defined "Poor" or "Fair" as worse health and the remaining categories as better health.

Additionally, participants were asked about their current symptoms at the time of the questionnaire. The respective symptom list consisted of 21 symptoms commonly linked to PCC. The list was compiled from various publications available at the time the questionnaire was developed (Table S1).

#### SARS-CoV-2 infections and post COVID-19 condition

Participants were subsequently asked whether they ever had a SARS-CoV-2 infection. If they had a previous infection, the participants were asked if they had any symptoms at four time points after their first positive test (during the acute infection, 2–3 months after infection, 4–12 months, and 1 year or more after infection). We defined PCC as the response "yes" to the question if they had any symptoms in the time window from 4 to 12 months after SARS-CoV-2 infection. Additionally, the same symptom list as for the current symptoms was used to ask about individual symptoms present at the respective time point. The number and date (months only) of vaccinations were also reported.

We defined three groups for this analysis: (1) individuals with no reported SARS-CoV-2 infection ("No infection"), (2) individuals who reported an infection and were not classified as PCC ("Reported infection, no PCC"), (3) individuals who reported an infection and were classified as PCC ("PCC"). We restricted the analysis to individuals who had reported only one infection at the time of the survey, multiple infections were rare and our questionnaire was not well suited to distinguish start of symptoms in case of multiple infections. Additionally, the infection had to have occurred at least four months prior to answering the survey (Figure S1).

Furthermore, we performed a sensitivity analysis that excluded individuals who were possibly misclassified as having PCC (s. File S1 for further explanations).

### Statistical analysis

We report frequencies and percentages to describe and compare the three infection/PCC groups. We descriptively compared self-perceived health of the groups and examined the proportion of individuals in each group reporting potentially PCC-related symptoms at the time of the survey. Mixed effects logistic regression was used to analyze the association between the number of current symptoms, infection/PCC group (as described above), and worse health. Good or better self-perceived health was used as the reference. The mixed model was used to account for possibly higher similarity within regional samples. In the first model, we studied the association between infection/PCC and worse health adjusted for age, sex, education and presence of any of the selected pre-existing chronic diseases. In the second model, we additionally adjusted for the number of current symptoms to assess whether PCC status is associated with worse self-perceived health in another way besides the assessed current symptoms. A direct effect of PCC persisting after the adjustment for the current symptoms would indicate that either not all relevant symptoms are considered, the current symptoms are differently perceived by participants classified as PCC or PCC is associated with (poorer) self-perceived health beyond observable symptoms. The 18 study centers were included as random effects in both models. In additional analyses, we used separate models for both sexes and used age groups instead of assuming a linear relationship with age. We also performed sensitivity analyses with additional definitions of PCC (File S1). All analyses were performed in R (Version 4.2.0) and odds ratios (OR) with 95% confidence intervals (95% CI) are reported for the regression analyses.

## Results

### Characteristics of the participants

Of the 150,722 invited participants, 110,375 (73%) completed the online survey, of which 86,833 (79%) fulfilled the criteria for analysis. About half of the sample reported a SARS-CoV-2 infection, of which 15,656 were classified as having developed PCC (37%) (Table 1). The mean age of the group with no infection was 57.9 years while it was 52.0 for the "infection, no PCC" group and 51.6 for the PCC group. Women were more likely to be classified as having PCC than men (Table S2).

**Table 1:** Participant characteristics (*N* = 86,833).

	*N*	%
Sex		
Male	42,844	49.3
Female	43,989	50.7
Age group		
20–29	2496	2.9
30–39	9824	11.3
40–49	14,169	16.3
50–59	26,844	30.9
60–69	21,246	24.5
70+	12,254	14.1
Education		
Low	969	1.1
Medium	30,577	9.5
High	52,471	35.2
Missing	2816	3.2
Study center		
Augsburg	8392	9.7
Regensburg	4302	5.0
Mannheim	4715	5.4
Freiburg	5387	6.2
Saarbrücken	4302	5.0
Essen	4398	5.1
Münster	4585	5.3
Düsseldorf	3767	4.3
Halle	3807	4.4
Leipzig	4253	4.9
Berlin Nord	5099	5.9
Berlin Mitte	5207	6.0
Berlin Süd	4870	5.6
Hannover	3532	4.1
Hamburg	4906	5.6
Bremen	5229	6.0
Kiel	3829	4.4
Neubrandenburg	6253	7.2

	*N*	%
Infection status and PCC		
No reported infection	44,451	51.2
Reported infection, no PCC	26,726	30.8
Reported infection, PCC	15,656	18.0
Number of reported vaccinations at time of survey		
None	3285	3.8
1	675	0.8
2	7315	8.4
3	56,103	64.6
4	16,482	19.0
5	605	0.7
I do not want to report it	997	1.1
Missing	1371	1.6
Relevant comorbidities		
No comorbidities	53,579	61.7
At least one comorbidity	32,147	37.0
Missing	1107	1.3

### Self-perceived health and symptoms by infection/PCC status

**Fig. 1:**
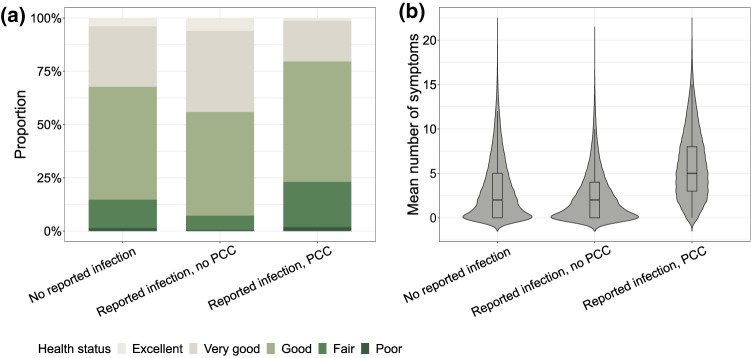
(**a**) Health status by infection/PCC group and (**b**) Mean number of current symptoms by infection/PCC group.

Overall, 86% of participants rated their health at least as "good". This percentage was similar among individuals with no reported infection (85%) and higher for those with infection but no PCC (93%). In contrast, those with PCC reported good health less frequently (77%) (Fig. 1a, Table S3). Only 4% of the PCC group reported no current symptoms. The median number of current symptoms reported by individuals classified as having PCC was 5 (interquartile range [IQR] = 5), compared to 2 in the "no infection" (IQR = 5) and the "infection and no PCC" group (IQR = 4, Fig. 1b, Figure S2). In both infection groups combined (with or without PCC), the median number of current symptoms was 3 (IQR = 5). In the PCC group, the median number of current symptoms was 6 for women and 5 for men (IQR = 5 for both groups, Figure S3). For the same number of current symptoms, the proportion of individuals with worse health was similar across the three groups (Figure S4).

**Fig. 2:**
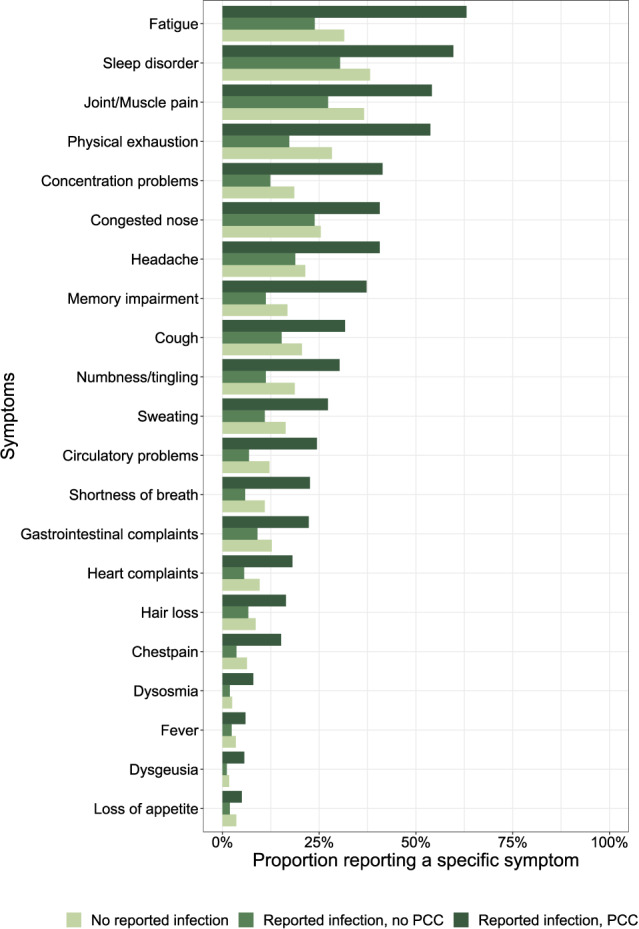
Proportion of participants reporting specific individual symptoms at the time of the survey by infection/PCC group sorted by overall frequency.

In the PCC group, the two most commonly reported current symptoms were fatigue (63%) and sleep disorder (60%) (Fig. 2), with 74% of individuals reporting at least one of these symptoms, compared to 46% in the "no infection" group and 38% in the "infection/no PCC" group.

### Association between infection/PCC group and self-reported health

There was an increased odds for individuals classified as having PCC to have worse self-perceived health (OR = 1.84, 95% CI = 1.75; 1.93), but not for those with reported infection but without PCC (OR = 0.55; 95% CI = 0.52; 0.59), compared to the group that did not report an infection, adjusted for age, sex, education, the selected comorbidities, and study center as random effect (Table 2). This estimate for the increase in the odds of worse self-perceived health is higher than the effect of an increase in age by 30 years (OR = 1.21^3 ^= 1.77). However, when the number of current symptoms was included in the model, the association between PCC status and worse self-perceived health disappeared (OR below 1). Each additional symptom increased the odds of having worse health by 41% (OR = 1.41, 95% CI = 1.40, 1.42). This estimate was somewhat higher than the effect of an increase in age by 10 years (OR = 1.32, 95% CI = 1.29, 1.34). The regression stratified by sex and the sensitivity analyses showed consistent results (Table S4 and File S1). The analysis using age groups instead of age as a linear term confirmed the linear relationship of age with the outcome (Table S5).

**Table 2:** Association between infection/PCC group and self-perceived health. Logistic regression, poor and fair versus good or better (reference) self-perceived health, *N* = 84,017.

	Model 1*		Model 2*	
	OR	95% CI	OR	95% CI
Age				
Per 10 years increase	1.21	1.19, 1.23	1.32	1.29, 1.34
Sex				
Male	Ref.		Ref.	
Female	1.22	1.17, 1.27	0.85	0.81, 0.89
Education				
High	Ref.		Ref.	
Medium	1.35	1.30, 1.41	1.20	1.14, 1.25
Low	1.85	1.58, 2.17	1.50	1.24, 1.80
Infection status and PCC				
No reported infection	Ref.		Ref.	
Reported infection, no PCC	0.55	0.52, 0.59	0.74	0.70, 0.79
Reported infection, PCC	1.84	1.75, 1.93	0.89	0.84, 0.94
Relevant comorbidities				
No comorbidities	Ref.		Ref.	
At least one comorbidity	2.89	2.77, 3.01	1.36	1.29, 1.42
Current symptoms				
Per one symptom increase	–	–	1.41	1.40, 1.42

* Mutually adjusted for all variables listed in the table, additionally adjusted for study center as random effect.

## Discussion

We found that the PCC group reported worse self-perceived health and a higher number of PCC-related symptoms at the time of the survey than the two comparison groups without PCC. The difference in self-perceived health was substantial (the OR somewhat more than for an increase in age by 30 years), and it appears to be fully explained by the number of current symptoms.

Other studies also found that individuals with PCC were more likely to report worse health. For example, a study in Italy observed lower SF-36 scores in their PCC population, when comparing the values to a national normative group^21^. Similarly, a recent study, using data from a large online survey in Germany, demonstrated that while adjusting for the general burden of the pandemic, individuals who experienced a SARS-CoV-2 infection had worse mental health than those who did not, the difference resulting from the group with PCC^22^.

Multiple studies have shown that the number of symptoms is associated with perceived health of individuals^23–25^. In one study, the number of symptoms was estimated to explain 35% of the variability in perceived health^23^. These studies used more general symptoms, however, some were also included in our analysis as they were considered to be linked to PCC. Our analysis suggests that the symptoms reported to be associated with PCC largely account for the reduced perceived health of those with PCC. PCC could also have a direct association with poorer self-perceived health (e.g. due to other unmeasured impairments from COVID-19 or as a precondition) apart from these symptoms, however, our results suggest that this is not the case. The effects of PCC on self-perceived health seem to be explained by the investigated symptoms. Although the pathogenesis of these symptoms is not fully understood, at least the effect of PCC on self-perceived health is measurable through these symptoms. Still, some symptoms may be more important in this relationship than others. Additionally, the symptom list was specifically created to ask about symptoms that are linked to PCC and might not be appropriate to study the association between perceived health and other diseases. The overcorrection of the effect (OR below 1) may be related to differences in symptom severity beyond the simple presence of symptoms, but we did not assess this aspect.

The symptoms reported most frequently by individuals classified as having PCC in our study were fatigue, sleep disorder, joint/muscle pain, and reduced exertion tolerance/quick physical exhaustion. While fatigue was also one of the most common symptoms in the two comparison groups analyzed, individuals classified as having PCC were more than twice as likely to report it. A meta-analysis also found that fatigue was reported by 58% of study participants with PCC^8^. The symptom profile found in PCC often resembles that of other unexplained post-acute infection syndromes and of ME/CFS^26^.

We found that the group with a reported infection without PCC rated their health better and had less current symptoms than the group without a reported infection. Various explanations of this observation are possible: either a preexisting difference between those with infection vs. not or a difference resulting from a selection among those experiencing infection. The group with no reported infection was older than the group reporting infection, possibly indicating that this group was better protected (by vaccination, restriction of social contacts, or a conscious protective behavior). At the same time, this means that individuals in this group were actually considered at a higher risk for a severe course of infection due to comorbidities and consequently had worse health and more symptoms than those who became infected. This is further complicated by the fact that self-perceived health is a subjective measure and those who survived infection without developing PCC can experience a positive reinforcement. We cannot distinguish between these effects without analyzing detailed data on pre-infection health status, thus some uncertainty remains.

Compared to previous studies investigating the symptom burden in the general population, the median number of symptoms was lower in our study in the "no infection" and the "infection/no PCC" group. One study reported a mean symptom number of 5.4 and another a median number of 5^10,11^. However, both of these studies used a list of symptoms that included twice as many as our study. In line with our findings, both of these studies reported that women had a higher symptom burden than men. In our study, women’s worse self-perceived health was also partly explained by the higher number of symptoms.

The strength of this analysis is the large population-based sample. At the same time, there are also several limitations. First, our data provide a snapshot with respect to the time of the pandemic and the proportions of PCC after infection with different SARS-CoV-2 variants. While we demonstrated that symptom patterns of PCC are similar for the known virus variants (with the exception of smell and taste loss, which was less common following infection with the omicron variant)^27^, there are differences for example in the probability of developing PCC^16^ or the time until recovery across virus variants^28^. Second, all information is based on self-reports, possibly leading to misclassification of some cases concerning the infection and PCC. It is possible that some participants were not aware that they experienced an infection. This could be particularly the case for asymptomatic or oligosymptomatic cases. These participants could still develop PCC symptoms, but not be aware of their origin. This would attenuate the effect of PCC. The classification as PCC was based on a positive response to the question whether the participant has/had any symptoms 4–12 months after infection. Although the question was meant to ask about symptoms related to the infection, some participants may have reported symptoms unrelated to the infection. However, given that we also asked about the beginning of symptoms, we could show that the majority of respondents (> 64%, see sensitivity analysis) reported symptoms that started after infection. We conducted an analysis restricted to this group and the results obtained from model 2 were virtually the same. In model 1, a change was to be expected, because the number of symptoms in the PCC group increased due to the change of the definition of PCC. This supports the assumption that most participants understood the question as it was intended. Another problem is that for the classification of PCC, participants had to retrospectively report their symptoms for the time window they might have already left. This could have led to an omission of PCC cases that recovered since, as their symptoms were less present to them.

We did not have information about clinical diagnoses of PCC to compare to our classification based on self-reported symptoms. The subjective nature of many of the symptoms is a diagnostic challenge, but this is not restricted to our study and it is an inherent issue of PCC. Still, the number of individuals classified as having PCC is high in our study. Furthermore, we did not assess possible treatments for PCC, which could have reduced the burden of symptoms in the PCC group. Our results are mostly limited to non-hospitalized individuals, as less than 1% of the study population was hospitalized^16^, thus health impairment due to PCC is probably underestimated. While the proportion of hospitalized SARS-CoV-2 cases at population level is also low, it is possible that NAKO participants with a stronger impairment after infection did not participate in the survey. In addition, we assessed only the presence of symptoms, not their severity. We limited the analysis to individuals with no or one reported infection to ensure a more homogenous group. It is not clear how the burden of PCC or the relationship with symptoms might change after multiple infections, and future studies should investigate this. Additionally, while self-perceived health is a valid and efficient measure of physical and mental health^29^, it could be beneficial to use multiple measures. For the description of health impairment in those with PCC, more specific instruments could be useful. Also, the assessment of baseline comorbidities was conducted between 2014 and 2019 which resulted in a considerable interval to the analyzed questionnaire for some participants. In this time period, new comorbidities might have developed. Further, some of the relevant comorbidities could not be conclusively represented by the questionnaire. Immunosuppression for example could only be approximated by having HIV/AIDS.

In conclusion, we found substantially worse self-perceived health among persons with PCC and the number of reported symptoms appeared to fully explain the impaired self-perceived health. This highlights the need for addressing symptoms among those suffering from post-infection syndromes along existing guidelines, as well as the need for more research to find causal treatments^26,30,31^.

## Electronic supplementary material

Below is the link to the electronic supplementary material.

Supplementary Material 1

Supplementary Material 2

## Data Availability

Data used for this analysis can be made available upon request to the corresponding author and to the Use and Access Committee of the NAKO. The request can be submitted via: https://transfer.nako.de/transfer/index.
