# Supplementary material for: Cross-sectional study of health impairment related to post COVID-19 condition among participants of a large population-based cohort in Germany
Source: Sci Rep. 2025 Jul 16;15:25830. doi: 10.1038/s41598-025-07894-7 (PMC12267414; doi:10.1038/s41598-025-07894-7)
Supplement: Supplementary file 1 — Supplementary Material 1 [file 41598_2025_7894_MOESM1_ESM.docx]

### **Sensitivity Analyses**

**1.**

**Methods**

As the primary definition for PCC relied on a single question, we likely misclassified some individuals as having PCC. Therefore, we performed a sensitivity analysis excluding these individuals.

Additionally to reporting if an individual had one out of 21 symptoms at the time of the survey, the participants were asked when this reported symptom started (Month and Year). We matched this information with the reported SARS-CoV-2 infection date and classified the start of the symptom as starting before or after the infection.

In the sensitivity analysis, an individual would only be classified as having PCC if they reported at least one current symptom that started after their infection. In addition, this symptom had also to be reported 4 to 12 months after their SARS-CoV-2 infection. If an individual did not fulfill the criteria, they were excluded from the analysis.

**Results**

In the sensitivity analysis, 9990 (64%) individuals of the initial 15656 were classified as having developed PCC. The regression analysis showed virtually the same results as the analysis without excluding certain individuals (Table).

Table – Sensitivity analysis of the logistic regression models reported in Table 2

|  |  | Model 1* | | Model 2* | |
| --- | --- | --- | --- | --- | --- |
|  |  | OR | 95% CI | OR | 95% CI |
| Age | |  |  |  |  |
|  | per 10 years increase | 1.22 | 1.20, 1.24 | 1.32 | 1.29, 1.35 |
| Sex | |  |  |  |  |
|  | Male | Ref. |  | Ref. |  |
|  | Female | 1.22 | 1.17, 1.27 | 0.85 | 0.81, 0.89 |
| Education | |  |  |  |  |
|  | High | Ref. |  | Ref. |  |
|  | Medium | 1.35 | 1.29, 1.41 | 1.20 | 1.14, 1.25 |
|  | Low | 1.83 | 1.57, 2.14 | 1.49 | 1.24, 1.79 |
| Infection status and PCC | |  |  |  |  |
|  | No reported infection | Ref. |  | Ref. |  |
|  | Reported infection, no PCC | 0.69 | 0.66, 0.73 | 0.79 | 0.75, 0.83 |
|  | Reported infection, PCC | 2.17 | 2.05, 2.29 | 0.87 | 0.82, 0.93 |
| Relevant comorbidities | |  |  |  |  |
| No comorbidities | | Ref. |  | Ref. |  |
| At least one comorbidity | | 2.93 | 2.81, 3.05 | 1.36 | 1.29, 1.42 |
| Current symptoms | |  |  |  |  |
|  | per one symptom increase | – | – | 1.42 | 1.41, 1.42 |
| * Mutually adjusted for all variables listed in the model, additionally adjusted for study center as random effect. | | | | | |

**2.**

**Methods**

We performed one sensitivity analysis where we only classified individuals as having PCC if the participants reported at least one out of five specific symptoms (Fatigue, physical exhaustion, concentration problems, memory impairment, or shortness of breath) 4 to 12 months after SARS-CoV-2 infection. If an individual did not fulfill the criteria, they were classified as not having PCC.

**Results**

In this sensitivity analysis, 11954 (76%) individuals of the initial 15656 were classified as having developed PCC. The regression analysis showed virtually the same results as the analysis without excluding certain individuals (Table).

Table – Sensitivity analysis of the logistic regression models reported in Table 2

|  |  | Model 1* | | Model 2* | |
| --- | --- | --- | --- | --- | --- |
|  |  | OR | 95% CI | OR | 95% CI |
| Age | |  |  |  |  |
|  | per 10 years increase | 1.22 | 1.20, 1.24 | 1.31 | 1.29, 1.34 |
| Sex | |  |  |  |  |
|  | Male | Ref. |  | Ref. |  |
|  | Female | 1.20 | 1.15, 1.25 | 0.85 | 0.81, 0.89 |
| Education | |  |  |  |  |
|  | High | Ref. |  | Ref. |  |
|  | Medium | 1.35 | 1.29, 1.41 | 1.20 | 1.14, 1.26 |
|  | Low | 1.84 | 1.57, 2.16 | 1.50 | 1.25, 1.81 |
| Infection status and PCC | |  |  |  |  |
|  | No reported infection | Ref. |  | Ref. |  |
|  | Reported infection, no PCC | 0.55 | 0.52, 0.58 | 0.72 | 0.68, 0.77 |
|  | Reported infection, PCC | 2.33 | 2.21, 2.46 | 0.94 | 0.89, 1.00 |
| Relevant comorbidities | |  |  |  |  |
|  | No comorbidities | Ref. |  | Ref. |  |
|  | At least one comorbidity | 2.80 | 2.69, 2.92 | 1.36 | 1.29, 1.42 |
| Current symptoms | |  |  |  |  |
|  | per one symptom increase | – | – | 1.41 | 1.40, 1.42 |
| * Mutually adjusted for all variables listed in the table, additionally adjusted for study center as random effect. | | | | | |
