# Supplementary material for: Cross-sectional study of health impairment related to post COVID-19 condition among participants of a large population-based cohort in Germany
Source: Sci Rep. 2025 Jul 16;15:25830. doi: 10.1038/s41598-025-07894-7 (PMC12267414; doi:10.1038/s41598-025-07894-7)
Supplement: Supplementary file 2 — Supplementary Material 2 [file 41598_2025_7894_MOESM2_ESM.docx]

## **Supplement**

### **Figures**


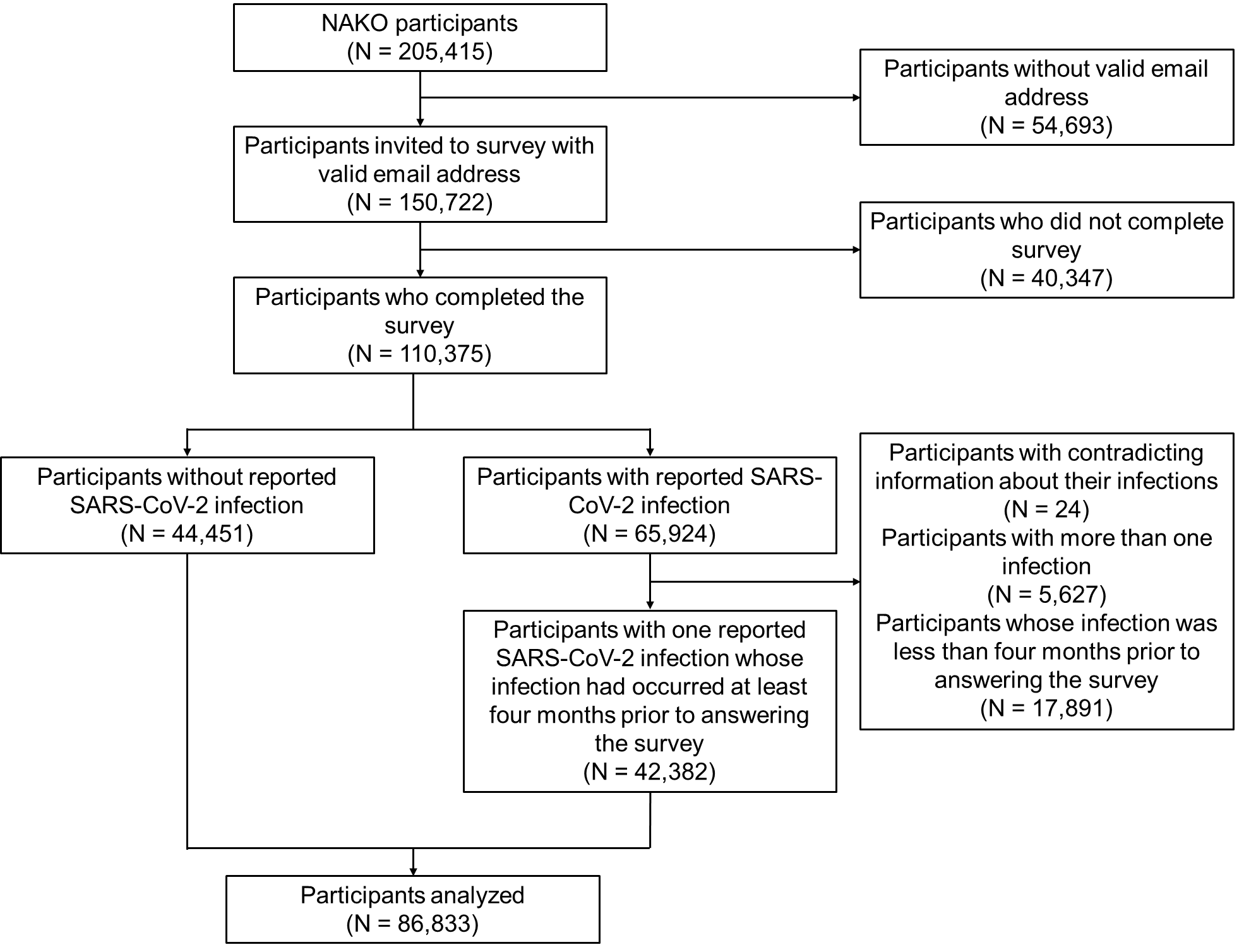


Figure S1 – Participant flowchart


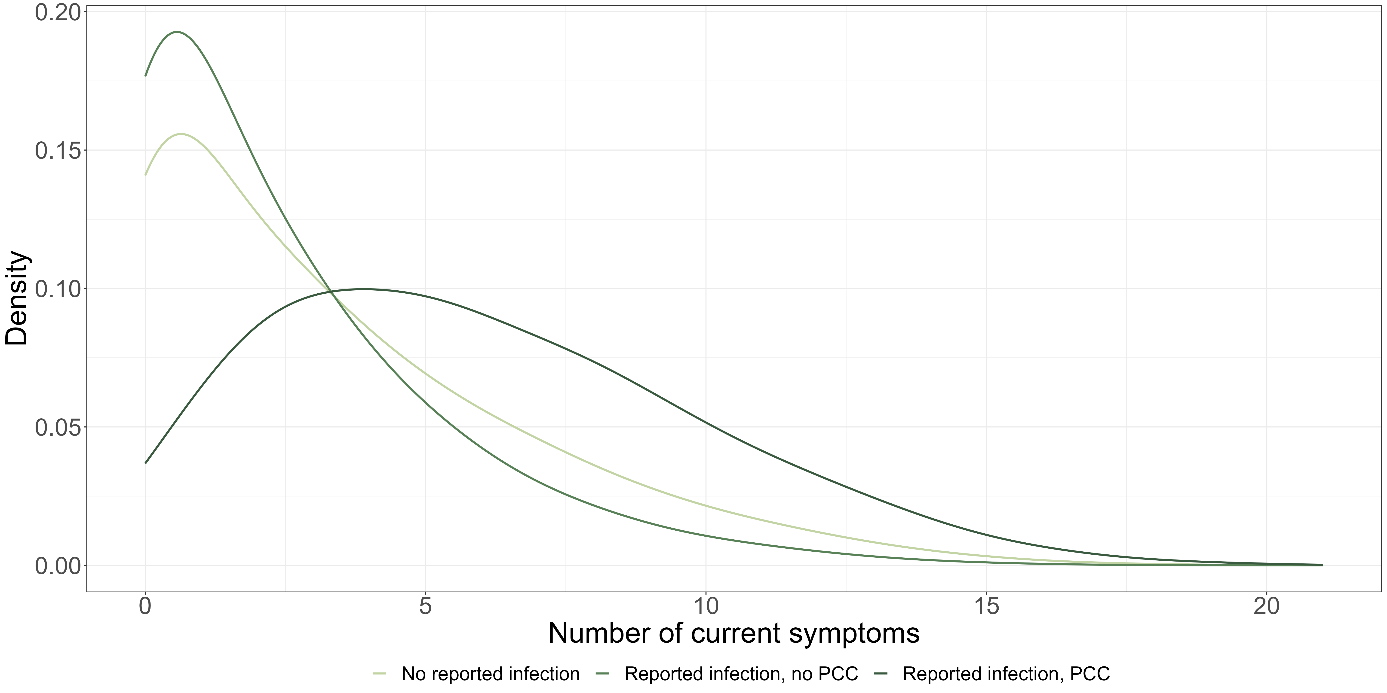


Figure S2 – Number of current symptoms by infection/PCC group

**
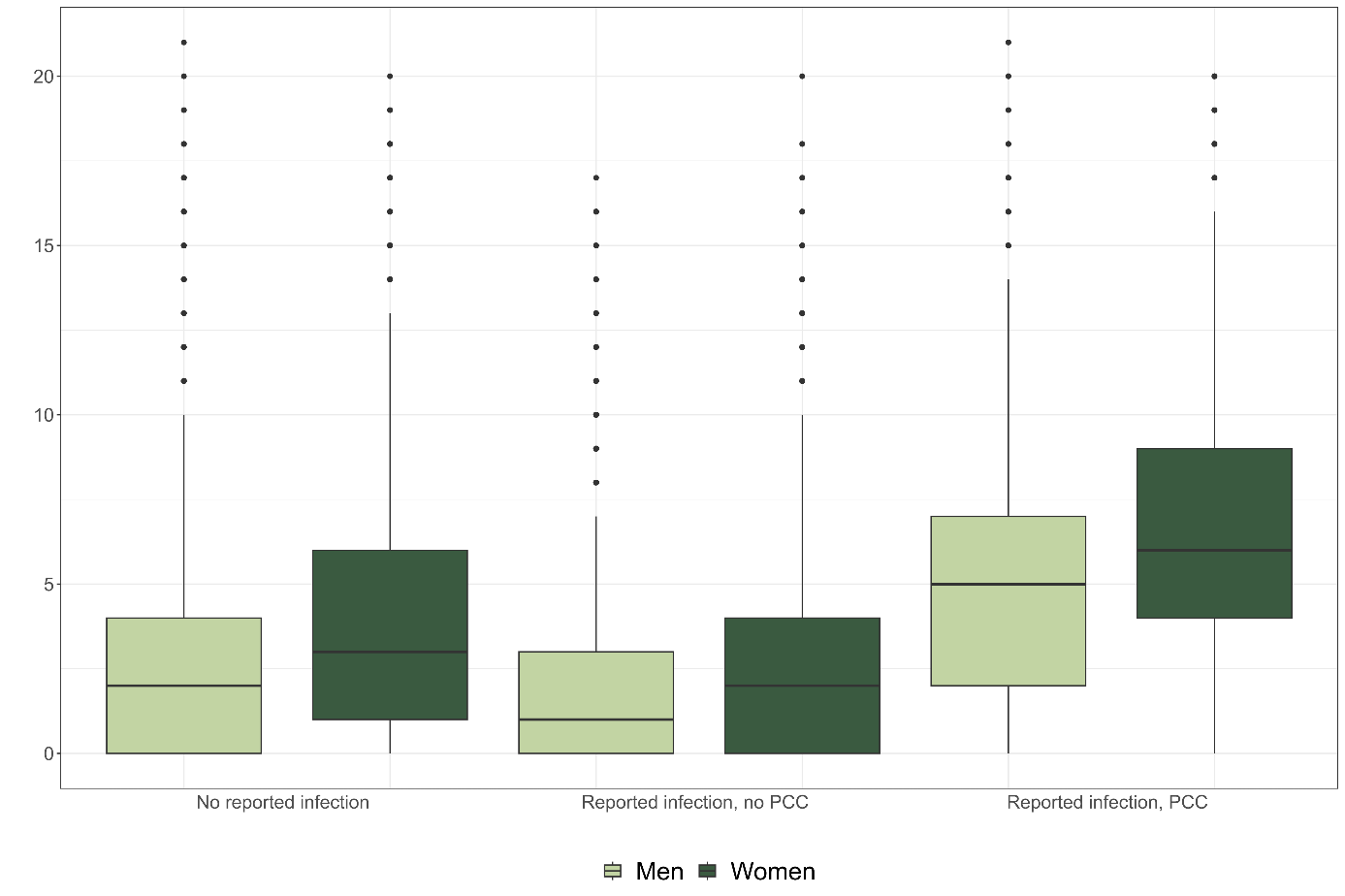
**

Figure S3 – Mean number of reported symptoms by infection/PCC group and sex


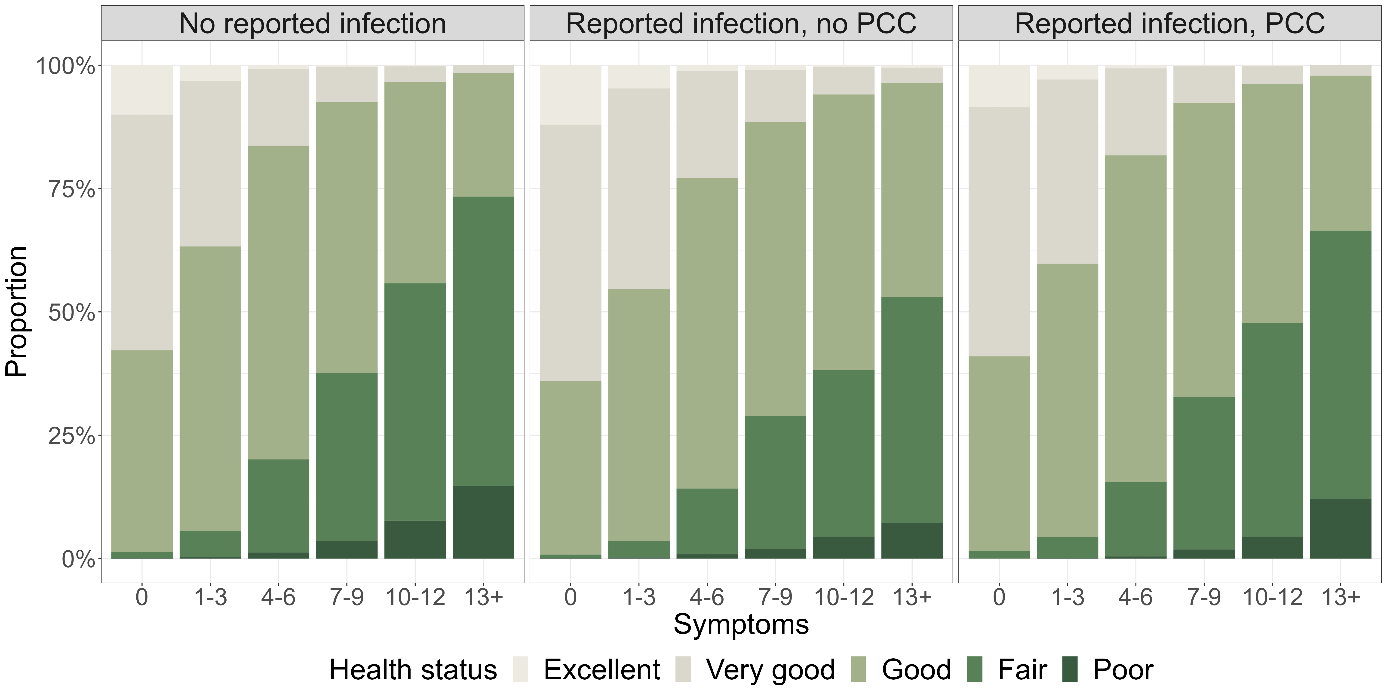


Figure S4 – Proportion of individuals with certain health status by symptom number and infection/PCC group

### **Tables**

Table S1 – Symptom list and publications used for the development

| Symptoms in final list | Fever |
| --- | --- |
|  | Dysosmia |
|  | Dysgeusia |
|  | Joint/Muscle pain |
|  | Fatigue |
|  | Sleep disorder |
|  | Physical exhaustion |
|  | Sweating |
|  | Memory impairment |
|  | Concentration problems |
|  | Headache |
|  | Congested nose |
|  | Cough |
|  | Shortness of breath |
|  | Chest pain |
|  | Heart complaints |
|  | Gastrointestinal complaints |
|  | Loss of appetite |
|  | Hair loss |
|  | Circulatory problems |
|  | Numbness/tingling |
|  | |
| Publications used to develop symptom list | Evans, R. A. et al. Clinical characteristics with inflammation profiling of long COVID and association with 1-year recovery following hospitalisation in the UK: a prospective observational study. The Lancet Respiratory Medicine vol. 10 761–775 (2022). |
|  | Gao, Y., Liang, W., Li, Y., He, J. & Guan, W. The Short- and Long-Term Clinical, Radiological and Functional Consequences of COVID-19. Archivos de Bronconeumología vol. 58 32–38 (2022). |
|  | Lopez-Leon, S. et al. More than 50 long-term effects of COVID-19: a systematic review and meta-analysis. Scientific Reports vol. 11 (2021). |
|  | Lorent, N. et al. Prospective longitudinal evaluation of hospitalised COVID-19 survivors 3 and 12 months after discharge. ERJ Open Research vol. 8 00004–02022 (2022). |
|  | Robineau, O. et al. Persistent symptoms after the first wave of COVID-19 in relation to SARS-CoV-2 serology and experience of acute symptoms: A nested survey in a population-based cohort. The Lancet Regional Health - Europe vol. 17 100363 (2022). |
|  | Silva, C. C. et al. Muscle dysfunction in the long coronavirus disease 2019 syndrome: Pathogenesis and clinical approach. Reviews in Medical Virology vol. 32 (2022). |
|  | Sugiyama, A. et al. Long COVID occurrence in COVID-19 survivors. Scientific Reports vol. 12 (2022). |
|  | Tegenthoff, M., Drechsel-Schlund, C. & Widder, B. Neurologisch-psychiatrische Begutachtung des Post-COVID-Syndroms. Der Nervenarzt vol. 93 804–811 (2022). |
|  | Whitaker, M. et al. Persistent COVID-19 symptoms in a community study of 606,434 people in England. Nature Communications vol. 13 (2022). |

Table S2 – Participant characteristics by infection/PCC group

|  |  | No reported infection | | Reported infection,  no PCC | | Reported infection,  PCC | |
| --- | --- | --- | --- | --- | --- | --- | --- |
|  |  | N | % | N | % | N | % |
|  |  | 44451 |  | 26726 |  | 15656 |  |
| Sex | |  |  |  |  |  |  |
|  | Male | 22148 | 49.8 | 14304 | 53.5 | 6392 | 40.8 |
|  | Female | 22303 | 50.2 | 12422 | 46.5 | 9264 | 59.2 |
| Age group | |  |  |  |  |  |  |
|  | 20-29 | 818 | 1.8 | 1116 | 4.2 | 562 | 3.6 |
|  | 30-39 | 3537 | 8.0 | 4020 | 15.0 | 2267 | 14.5 |
|  | 40-49 | 5471 | 12.3 | 5300 | 19.8 | 3398 | 21.7 |
|  | 50-59 | 12891 | 29.0 | 8532 | 31.9 | 5421 | 34.6 |
|  | 60-69 | 13034 | 29.3 | 5279 | 19.8 | 2933 | 18.7 |
|  | 70+ | 8700 | 19.6 | 2479 | 9.3 | 1075 | 6.9 |
| Education | |  |  |  |  |  |  |
|  | Low | 587 | 1.3 | 239 | 0.9 | 143 | 0.9 |
|  | Medium | 16387 | 36.9 | 8660 | 32.4 | 5530 | 35.3 |
|  | High | 26630 | 59.9 | 16632 | 62.2 | 9209 | 58.8 |
|  | Missing | 847 | 1.9 | 1195 | 4.5 | 774 | 4.9 |
| Study center | |  |  |  |  |  |  |
|  | Augsburg | 4081 | 9.2 | 2812 | 10.5 | 1499 | 9.6 |
|  | Regensburg | 2033 | 4.6 | 1447 | 5.4 | 822 | 5.3 |
|  | Mannheim | 2437 | 5.5 | 1436 | 5.4 | 842 | 5.4 |
|  | Freiburg | 2643 | 5.9 | 1744 | 6.5 | 1000 | 6.4 |
|  | Saarbrücken | 2292 | 5.2 | 1227 | 4.6 | 783 | 5.0 |
|  | Essen | 2377 | 5.3 | 1258 | 4.7 | 763 | 4.9 |
|  | Münster | 2350 | 5.3 | 1450 | 5.4 | 785 | 5.0 |
|  | Düsseldorf | 1876 | 4.2 | 1239 | 4.6 | 652 | 4.2 |
|  | Halle | 1922 | 4.3 | 1143 | 4.3 | 742 | 4.7 |
|  | Leipzig | 2055 | 4.6 | 1361 | 5.1 | 837 | 5.3 |
|  | Berlin Nord | 2666 | 6.0 | 1451 | 5.4 | 982 | 6.3 |
|  | Berlin Mitte | 2660 | 6.0 | 1612 | 6.0 | 935 | 6.0 |
|  | Berlin Süd | 2490 | 5.6 | 1468 | 5.5 | 912 | 5.8 |
|  | Hannover | 1935 | 4.4 | 1037 | 3.9 | 560 | 3.6 |
|  | Hamburg | 2565 | 5.8 | 1491 | 5.6 | 850 | 5.4 |
|  | Bremen | 2949 | 6.6 | 1455 | 5.4 | 825 | 5.3 |
|  | Kiel | 2084 | 4.7 | 1098 | 4.1 | 647 | 4.1 |
|  | Neubrandenburg | 3036 | 6.8 | 1997 | 7.5 | 1220 | 7.8 |
|  | None | 1226 | 2.8 | 1447 | 5.4 | 612 | 3.9 |
| Number of reported vaccinations at time of survey | |  |  |  |  |  |  |
|  | 1 | 111 | 0.2 | 321 | 1.2 | 243 | 1.6 |
|  | 2 | 2152 | 4.8 | 3013 | 11.3 | 2150 | 13.7 |
|  | 3 | 26501 | 59.6 | 18542 | 69.4 | 11060 | 70.6 |
|  | 4 | 12520 | 28.2 | 2703 | 10.1 | 1259 | 8.0 |
|  | 5 | 517 | 1.2 | 62 | 0.2 | 26 | 0.2 |
|  | I do not want to report it | 461 | 1.0 | 389 | 1.5 | 147 | 0.9 |
|  | Missing | 963 | 2.2 | 249 | 0.9 | 159 | 1.0 |
| Relevant comorbidities | |  |  |  |  |  |  |
|  | No comorbidities | 27091 | 60.9 | 18362 | 68.7 | 8126 | 51.9 |
|  | At least one comorbidity | 17149 | 38.6 | 7847 | 29.4 | 7151 | 45.7 |
|  | Missing | 211 | 0.5 | 517 | 1.9 | 379 | 2.4 |

Table S3 – Health status by infection/PCC group

|  |  | No reported infection | | Reported infection,  no PCC | | Reported infection,  PCC | |
| --- | --- | --- | --- | --- | --- | --- | --- |
|  |  | N | % | N | % | N | % |
|  |  | 44451 |  | 26726 |  | 15656 |  |
| Self-rated health | |  |  |  |  |  |  |
|  | Good or better | 37747 | 84.9 | 24714 | 92.5 | 12005 | 76.7 |
|  | Poor and fair | 6503 | 14.6 | 1926 | 7.2 | 3602 | 23.0 |
|  | Missing | 201 | 0.4 | 86 | 0.3 | 49 | 0.3 |

Table S4 – Association between Infection/PCC group and self-rated health. Logistic regression, poor and fair versus good or better (reference) self-rated health stratified by sex

|  |  |  | Men (N = 41,543) | | Women (N = 42,474) | |
| --- | --- | --- | --- | --- | --- | --- |
|  |  |  | OR | 95% CI | OR | 95% CI |
| Model 1* | | |  |  |  |  |
|  | Age | |  |  |  |  |
|  |  | per 10 years increase | 1.25 | 1.22, 1.29 | 1.18 | 1.16, 1.21 |
|  | Education | |  |  |  |  |
|  |  | High | Ref. |  | Ref. |  |
|  |  | Medium | 1.47 | 1.38, 1.56 | 1.29 | 1.22, 1.36 |
|  |  | Low | 1.95 | 1.47, 2.59 | 1.82 | 1.51, 2.20 |
|  | Infection status and PCC | |  |  |  |  |
|  |  | No reported infection | Ref. |  | Ref. |  |
|  |  | Reported infection, no PCC | 0.57 | 0.52, 0.62 | 0.54 | 0.50, 0.59 |
|  |  | Reported infection, PCC | 1.86 | 1.72, 2.01 | 1.82 | 1.71, 1.94 |
|  | Relevant comorbidities | |  |  |  |  |
|  |  | No comorbidities | Ref. |  | Ref. |  |
|  |  | At least one comorbidity | 2.79 | 2.62, 2.97 | 2.95 | 2.79, 3.12 |
| Model 2* | | |  |  |  |  |
|  | Age | |  |  |  |  |
|  |  | per 10 years increase | 1.34 | 1.30, 1.38 | 1.30 | 1.26, 1.34 |
|  | Education | |  |  |  |  |
|  |  | High | Ref. |  | Ref. |  |
|  |  | Medium | 1.27 | 1.18, 1.37 | 1.14 | 1.08, 1.22 |
|  |  | Low | 1.53 | 1.10, 2.12 | 1.48 | 1.18, 1.85 |
|  | Infection status and PCC | |  |  |  |  |
|  |  | No reported infection | Ref. |  | Ref. |  |
|  |  | Reported infection, no PCC | 0.75 | 0.69, 0.82 | 0.73 | 0.67, 0.80 |
|  |  | Reported infection, PCC | 0.88 | 0.81, 0.96 | 0.90 | 0.84, 0.97 |
|  | Relevant comorbidities | |  |  |  |  |
|  |  | No comorbidities | Ref. |  | Ref. |  |
|  |  | At least one comorbidity | 1.35 | 1.26, 1.45 | 1.36 | 1.27, 1.45 |
|  | Current symptoms | |  |  |  |  |
|  |  | per 1 Symptom increase | 1.44 | 1.42, 1.45 | 1.39 | 1.38, 1.41 |
| * mutually adjusted for all variables listed in the model, additionally adjusted for the NAKO study center as random effect | | | | | | |

Table S5 – Association between Infection/PCC group and self-rated health. Logistic regression, poor and fair versus good or better (reference) self-rated health using age groups instead assuming a linear relationship, N = 84,017.

|  |  | Model 1* | | Model 2* | |
| --- | --- | --- | --- | --- | --- |
|  |  | OR | 95% CI | OR | 95% CI |
| Age | |  |  |  |  |
|  | 20-29 | Ref. |  | Ref. |  |
|  | 30-39 | 1.26 | 1.01, 1.58 | 1.26 | 0.98, 1.60 |
|  | 40-49 | 1.63 | 1.32, 2.03 | 1.66 | 1.31, 2.10 |
|  | 50-59 | 2.14 | 1.73, 2.64 | 2.18 | 1.73, 2.75 |
|  | 60-69 | 2.36 | 1.91, 2.91 | 2.83 | 2.24, 3.58 |
|  | 70+ | 2.69 | 2.17, 3.34 | 3.51 | 2.77, 4.44 |
| Sex | |  |  |  |  |
|  | Male | Ref. |  | Ref. |  |
|  | Female | 1.22 | 1.17, 1.27 | 0.85 | 0.81, 0.89 |
| Education | |  |  |  |  |
|  | High | Ref. |  | Ref. |  |
|  | Medium | 1.35 | 1.30, 1.41 | 1.19 | 1.14, 1.25 |
|  | Low | 1.86 | 1.59, 2.18 | 1.50 | 1.24, 1.80 |
| Infection status and PCC | |  |  |  |  |
|  | No reported infection | Ref. |  | Ref. |  |
|  | Reported infection, no PCC | 0.55 | 0.52, 0.58 | 0.73 | 0.69, 0.78 |
|  | Reported infection, PCC | 1.81 | 1.72, 1.90 | 0.88 | 0.83, 0.94 |
| Relevant comorbidities | |  |  |  |  |
|  | No comorbidities | Ref. |  | Ref. |  |
|  | At least one comorbidity | 2.89 | 2.78, 3.02 | 1.36 | 1.30, 1.43 |
| Current symptoms | |  |  |  |  |
|  | per one symptom increase | – | – | 1.41 | 1.40, 1.42 |
| * Mutually adjusted for all variables listed in the table, additionally adjusted for study center as random effect. | | | | | |
